# Densification of MXene films by sequential bridging

**DOI:** 10.1093/nsr/nwab195

**Published:** 2021-10-28

**Authors:** Hui-Ming Cheng

**Affiliations:** Shenzhen Institute of Advanced Technology, Chinese Academy of Sciences, China; Shenyang National Laboratory for Materials Science, Institute of Metal Research, Chinese Academy of Sciences, China

Voids are an essential scientific issue when it comes to traditional composite materials, because they greatly degrade the latter's properties and usually cause catastrophic failure [1]. Many characterization methods, such as microscale X-ray computed tomography and ultrasonic attenuation, have been developed to detect the void microstructure in composites [1]. However, because nanoscale voids cannot be observed by the above methods, nanocomposites are usually considered compact materials. Although fractured samples of nanocomposites are often used to characterize their microstructure, the true microstructure cannot be observed from them [2–4]. Thus, void microstructure has long been ignored in assembled nanocomposites.

**Figure 1.**
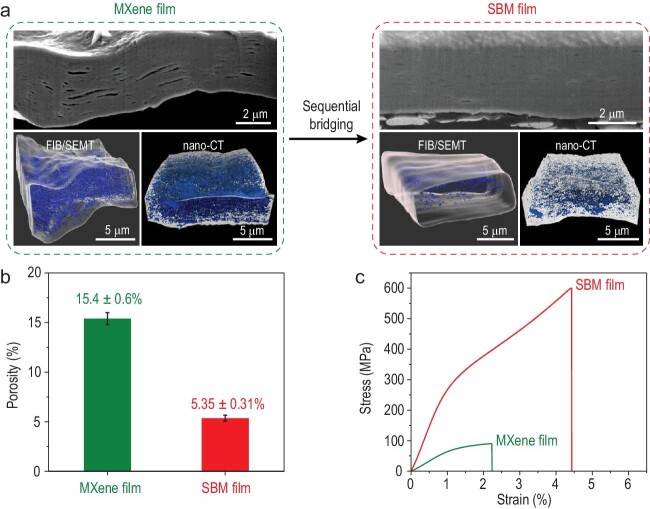
(a) SEM images of cross sections cut by an FIB and the 3D-reconstructed void microstructure derived from FIB/SEMT and nano-CT for unbridged MXene and SBM films [5]. (b) Porosity of the unbridged MXene and SBM films derived from density measurements [5]. (c) Typical tensile stress-strain curves of the unbridged MXene and SBM films [5]. Copyright © 2021, The American Association for the Advancement of Science.

In a landmark study recently published in *Science* [5] by Prof. Qunfeng Cheng's group from Beihang University, the three-dimensional (3D) void microstructure of titanium carbide MXene films was systematically characterized using focused ion beam/scanning electron microscopy tomography (FIB/SEMT) and nanoscale X-ray computed tomography (nano-CT), as shown in Fig. 1a. The FIB/SEMT and nano-CT results consistently demonstrate that the MXene films have numerous voids between adjacent platelets, with the volume of each void ranging from 2 × 10^–5^ to 1.5 μm^3^. The porosity of the MXene films is 15.4 ± 0.6%, as shown in Fig. 1b.

Cheng *et al*. [5] developed a simple and effective densification strategy using a sequential bridging process of hydrogen and covalent bonding. A hydrogen bonding agent (sodium carboxymethyl cellulose) fills and closes large-scale voids between MXene platelets, and a covalent bonding agent (borate ions) bridges adjacent MXene platelets bringing them closer and eliminating small-scale voids. As a result, the sequentially bridged MXene (SBM) films have very compact platelet stacking. FIB/SEMT and nano-CT (Fig. 1a) show that the void volume (5.35 ± 0.31%, as shown in Fig. 1b) in the SBM films is greatly decreased compared with that of the unbridged MXene films.

Because of this densified microstructure, and improved interlayer interactions, the SBM films have a much higher tensile strength, Young's modulus and toughness than the unbridged MXene films (Fig. 1c). The tensile strength and toughness of the SBM films are 583 ± 16 MPa and 15.9 ± 1.0 MJ/m^3^, respectively, which greatly exceed those of previously reported MXene-based films. Moreover, the SBM films show a much higher resistance to sonication damage, cyclic mechanical deformation, oxidation and stress relaxation than the unbridged films. Furthermore, the SBM films have excellent electrical conductivity and electromagnetic interference shielding performance.

In short, Cheng and co-workers [5] provided a milestone study in the field of nanocomposites, the key point of which is detecting and closing the voids that have been neglected in 2D assemblies. The results of this pioneering study may replace the previous densely stacked structure model of 2D assemblies and be of wide interest for academia and industry. In addition, closing voids by sequentially introducing hydrogen and covalent bonding agents provides a new strategy for assembling other 2D platelets into high-performance materials. It may also provide new ways of exploring nanocomposites from the viewpoint of voids, including their formation, characterization and effect on the material's performance.

***Conflict of interest statement*.** None declared.
